# Addressing barriers to care for African American men facing prostate cancer: a scoping review of navigation programs

**DOI:** 10.1007/s00520-025-10009-7

**Published:** 2025-10-14

**Authors:** Ashley Nicole Smith, Brittany A. Campbell, Ghilamichael Andemeskel, Peggy Tahir, Joseph Egbunikeokye, Tisha M. Felder, Barbara Cicerelli, Nynikka R. Palmer

**Affiliations:** 1 Division of General Internal Medicine, Department of Medicine, Zuckerberg San Francisco General Hospital and Trauma Center, University of California San Francisco, San Francisco, CA USA; 2 Action Research Center for Health, Division of Health and Society, Zuckerberg San Francisco General Hospital and Trauma Center, University of California San Francisco, San Francisco, CA USA; 3 Office of Community Engagement, Helen Diller Family Comprehensive Cancer Center, University of California San Francisco, San Francisco, CA USA; 4 University of California San Francisco Library, University of California San Francisco, San Francisco, CA USA; 5 School of Nursing, Community Health Systems, University of California San Francisco, San Franciso, CA USA; 6 College of Nursing, University of South Carolina, Columbia, SC USA; 7 Cancer Navigation Program, Department of Public Health, Zuckerberg San Francisco General Hospital and Trauma Center, San Francisco, CA USA; 8 Department of Urology, University of California San Francisco, San Francisco, USA

**Keywords:** Patient navigation, Prostate cancer, African American, Health disparities, Scoping review

## Abstract

**Purpose:**

African American/Black men are disproportionately impacted by prostate cancer (PCa). Patient navigation is an evidence-based approach to address barriers to care, improve access to care and health outcomes, and reduce disparities. This scoping review provides an in-depth examination of navigation programs in PCa care across the cancer continuum, with a focus on African American/Black men in the United States.

**Methods:**

We conducted a comprehensive literature search through September 1st, 2023, in PubMed, Embase, Web of Science, and CINAHL Complete, using keywords and index terms within three main themes: PCa, patient navigation, and African American/Black men. We included studies that described or investigated navigation programs/interventions for PCa from screening through survivorship and included at least 30% African American/Black men.

**Results:**

Of the 3,556 articles identified, 8 were included. Two articles covered the same navigation program—one reported the protocol and one reported quasi-experimental trial results. All but one study was conducted prior to 2012. The most common navigation activities reported were care coordination, education/information provision, and comfort/emotional support. Navigation improved screening uptake, PCa management, and access to supportive services. Only 3 articles provided information on navigation training. Both clinical (e.g., nurses) and non-clinical (e.g., peers) navigators were reported. Only 1 article discussed cultural tailoring to African American/Black men.

**Conclusion:**

Navigation programs in PCa care are beneficial; however, few studies were identified despite disease burden and disparities among African American/Black men. Contemporary navigation programs tailored for African American/Black men are needed to address persistent disparities.

**Supplementary Information:**

The online version contains supplementary material available at 10.1007/s00520-025-10009-7.

## Introduction

Prostate cancer (PCa) remains one of the most commonly diagnosed cancers among men in the United States (US) [1]. African American/Black (AAB) men are disproportionately burdened by PCa with higher incidence rates and likelihood of advanced disease at diagnosis, less likely to receive guideline-concordant care, report poorer satisfaction with care, and have more than twice the mortality rate compared to White men [2, 3]. Despite overall declines in PCa mortality due to improvements in treatment, significant racial disparities persist in quality of care and health outcomes [4]. Patient navigation, an evidence-based intervention designed to eliminate barriers to care and improve health outcomes, is an effective approach to reduce cancer disparities [5].

Patient navigation utilizes a patient-centered approach to resolve barriers to equitable care, ensure timely diagnostic care, and improve healthcare access [5]. Conceptualized by Dr. Harold Freeman in 1990, patient navigation was first developed and implemented in Harlem, New York to address racial disparities among low-income minority women with abnormal breast cancer screening results [6]. In 2012, the American College of Surgeons Commission on Cancer mandated navigation be part of all comprehensive cancer programs by 2015 [7]. Navigation has been used to reduce disparities among medically underserved populations, including racial and ethnic minorities and safety net populations across the cancer continuum [8, 9]. Systematic reviews report navigation increases participation in cancer screenings [8, 10], reduces hospital readmission, and improves quality of life and patient satisfaction with care [8, 11]. Navigation has also become a standard of care at many institutions [12] and has been adapted to address a range of health and social conditions for use in clinical and community-based settings [13].

One of the largest patient navigation studies included a partnership between the National Cancer Institute and the American Cancer Society to examine the impact of navigation on cancer care [9]. Navigation improved time to resolution of abnormal screening tests and treatment initiation; however, only 12% of the sample represented PCa [14] and even less were AAB men. As patient navigation was launched over 3 decades ago, we aimed to assess the current state of navigation programs in PCa care for AAB men, given the persistent disparities in quality of care and health outcomes, which represents one of the greatest disparities in cancer [15]. Our team systematically reviewed navigation programs across the PCa continuum to examine details on protocols and procedures, outcomes, training, management, and cultural context for AAB men. Results will identify gaps in the field and inform development and implementation of future navigation programs for PCa care to achieve health equity for underserved AAB men.

## Methods

We chose a scoping review methodology to provide a comprehensive overview of existing literature on patient navigation in PCa care, with a focus on AAB men. Scoping reviews are appropriate when systematically identifying, mapping, examining, and reporting specific characteristics and/or concepts in the literature [16, 17]. To our knowledge, this is the first review to examine components and processes of navigation programs, including cultural context, that focus on addressing barriers to care for AAB men facing PCa. This scoping review was conducted in adherence with the Preferred Reporting Items for Systematic reviews and Meta-Analyses extension for Scoping Reviews [18]. Our protocol was registered with the International Prospective Register of Systematic Reviews (PROSPERO; CRD42021221412) [19] and previously published [20].

### Inclusion and exclusion criteria

For inclusion, studies had to describe or investigate navigation programs or interventions for PCa at any point across the cancer control continuum (screening through survivorship) [21]. We defined navigation as any formal or informal, structured process designed to help patients overcome barriers to effective health care [22]. We searched for all study design types and methodologies. Included studies detailed navigation programs, intervention methods, or specific details on program development, content, type of navigators and their training, management, implementation, outcomes, or evaluation processes. Specifically, articles were eligible for inclusion if they met the following criteria: 1) included adult males of eligible screening age for PCa (≥ 40 years old) [23], 2) included a study population within the US, and 3) contained a sample of at least 30% AAB men. We chose a minimum of 30% AAB men for inclusion to ensure some consideration of the persistent disparities experienced by AAM men. Articles were excluded if the navigation program or intervention was designed for non-cancer conditions, focused on education or awareness only, or was an abstract only. We also excluded articles with programs or interventions that did not provide any follow-up after screening (e.g., test results or diagnostic resolution). We did not exclude articles based on study design, intervention, outcome measures, publication date, or quality.

### Information sources and search strategy

Our biomedical research librarian (PT) developed a search strategy to identify relevant studies using the following terms: PCa, patient navigation, and African American or Black men/males. We also included the terms: “health education,” “health promotion,” and “outreach programs” to capture studies synonymous with “navigation.” We conducted a comprehensive search of the following reference databases: PubMed, Web of Science, Embase, and Cumulative Index to Nursing and Allied Health (CINAHL) using the noted keywords and index terms (MESH and Emtree). See Supplementary Information for our search strategy. There was no restriction on dates to obtain a complete range of published studies. The database search was inclusive of all literature through September 1 st, 2023.

### Gray literature search

We developed and conducted a gray literature search using the following strategies: 1) handsearching references of included studies, 2) specialized Google searches, and 3) targeted web search of relevant professional organizations.

We hand searched the list of references of each study included, looking for relevant articles aligned with our inclusion criteria. Our research librarian developed specialized Google searches to identify potentially relevant materials using multiple combinations of our original search terms (i.e., “PCa,” “navigation,” and “AAB men”), within three domains of the Google search engine. This included “site:.edu” to yield materials unique to universities or relevant curriculums; “site:.org” to capture white papers, guidelines, or data from non-governmental agencies; and “site:.gov” to identify relevant government documents or reports. All searches were documented including dates performed.

### Screening and selection

The review and screening process involved two phases: 1) title and abstract review, and 2) full-text review. Title and abstract screenings were conducted independently by four team members (ANS, BAC, GA, and JE), where one lead reviewed all articles (ANS) and the other three were secondary reviewers. Any articles missing abstracts were removed from the screening process until an abstract or full text could be retrieved via additional searching or a request to the corresponding author. Articles lacking sufficient information for review in the title or abstract were moved to full-text review. If there was a disagreement on inclusion of an abstract between reviewers, we held team discussions to reach agreement. If an agreement was not reached, the article was moved to full-text review. Similarly, reviewers conducted the full-text review with one lead reviewer for all articles, and a secondary reviewer. When unable to reach consensus for inclusion, disagreements were resolved by a third reviewer – a senior researcher with experience conducting reviews (NRP).

**Fig. 1:**
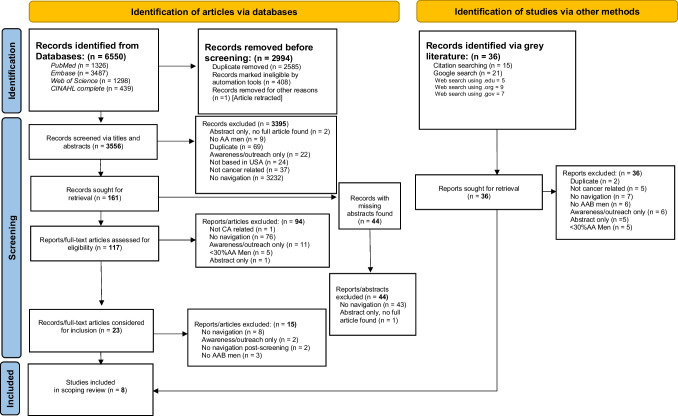
PRISMA flowchart describing search process for scoping review on patient navigation for African American men and prostate cancer

The most common reason for excluding an article at all phases of review (abstract and full-text review) was no patient navigation. See our PRISMA flowchart (Fig. 1) for an overview of information screening process. A total of 3,395 articles were excluded during the title and abstract review phase, primarily due to no patient navigation (n = 3232). Ninety-four articles were excluded during full-text review phase, also primarily due to no patient navigation (n = 76). And after extensive review and team discussions, 15 articles were also excluded during final review (n = 8 no patient navigation; n = 3 no AAB men; n = 2 no navigation beyond screening; n = 2 awareness only).

### Data extraction

We created a data extraction form and pilot tested it with all team members, where each reviewer independently reviewed a sample article that would be included to capture relevant data and compare our results. The data extraction form was iteratively revised based on feedback during team discussions. Following finalization, we used Research Electronic Data Capture (REDCap)—a secure, web-based system, for data collection and management [24]. We extracted data from included studies based on metrics set by the National Cancer Institute Patient Navigation Research Program [9] and knowledge of existing patient navigation programs. See our published protocol for additional details on data elements [20]. Data extraction categories included: reference, study information, target population, intervention/navigation program, data collection methods/measurements, navigator training and management, and cultural context. Similar to our study selection process, data extraction was completed independently by a primary (ANS) and secondary reviewer (BAC, GA, or JE), and disagreements were resolved through consensus discussions or a third reviewer (NRP). As a team, we synthesized our findings employing a narrative approach to summarize the results.

### Quality appraisal

We assessed all studies included for quality, although not required for scoping reviews [18], using the Mixed Methods Appraisal Tool Version 2018 (MMAT) [25]. We deemed the MMAT most appropriate for appraisal of methodological quality, given our review was not focused on any particular study design. All included studies were screened against five quality criteria questions per study design (qualitative, quantitative randomized controlled trials, quantitative non-randomized, quantitative descriptive, mixed methods). MMAT uses a categorical scale (yes, no, and cannot tell) for each quality criteria question to determine if criteria were met, and overall ratings were converted to percentages. Studies were assessed by counting the total number met out of the 5 total criteria, with a final score reported from 20 to 100% for quality criteria met [26]. Studies were scored as follows: (i) 20% indicates 1 criterion was met, (ii) 40% indicates 2 criteria were met, (iii) 60% refers to 3 criteria met, (iv) 80% refers to 4 criteria met, and (v) 100% indicates all 5 quality criteria were met. Two reviewers assessed the quality criteria for each included study. The team worked in pairs to resolve conflicts through discussion, when necessary. While no articles were excluded from the review based on scoring from the appraisal, scores provided an overview of study quality.

## Results

A total of 6,550 potentially eligible articles were identified from the search. After removing duplicate articles, including 1 retracted paper, 3,556 unique articles were screened based on title and abstract, with 161 articles undergoing full-text review. We had 84% reviewer agreement (2996/3556) for title and abstract review prior to team discussions. Of the 161 articles reviewed, 44 were missing abstracts. Articles with missing abstracts were located and underwent a full-text review. One additional paper was identified [27] after searching for records missing full text, resulting in the replacement of an abstract reporting on the same study [28]. This article was not identified in our original search as it outlined “minority groups” (indicated as one of the MeSH terms) whereas our search specified AAB men. Our search of relevant professional organizations yielded the following sites: Academy of Oncology of Nurse and Patient Navigators, National Navigation Roundtable, and The Patient Navigator Research Program. No relevant materials/records were identified from the gray literature search.

For full-text review, we had 80% reviewer agreement (129/161) prior to team discussions. A final total of 8 articles met our eligibility criteria and were chosen for inclusion in the scoping review (see Fig. 1). We had a median MMAT score of 90% for quality appraisal, with most studies meeting three or more of the five quality criteria. Specifically, one article met 1 of 5 criteria (20%) [29], one article met 3 of 5 criteria (60%) [30], two articles met 4 criteria (80%) [27, 31], and four articles met all 5 criteria (100%) [32–35].

### Study and intervention characteristics

The study characteristics reported in each article are presented in Table 1. The included studies reported a range of objectives. One study focused on increasing participation in an early detection program [34], two studies assessed the feasibility of support-based interventions [29, 33], while four studies evaluated the efficacy or impact of an intervention/program [30–32, 35]. Another study [27] covered the same navigation program – reporting the research protocol of a quasi—experimental trial [31].

**Table 1:** Characteristics of included studies (*n* = 8)

Author(s) & Year published	Study objective/design	Geographic location and setting	Study period	Sample & % African American/Black	Phase of cancer continuum*	Study outcomes	Navigation domains**
Powell et al., 1995	Increase participation of AAB men in a PCa early detection program, the Detroit Education and Early Detection Program (DEED) • Descriptive study	• Detroit, MI • Local Black churches	1993—1994	Sample size not reported100% AAB	Early Detection	• > 1000 men participated in DEED by June 1994 • 647 men screened by the end of the first year of DEED • 8% of men screened had elevated PSA levels • PCa detection rate was 2.5%	• Care coordination • Education/information provision • Comfort/emotional support • Direct care provision • Financial assistance
Weinrich et al., 1998	Measure the effects of four educational interventions on free PCa screening including client-navigation • Quasi-experimental 2 × 2 factorial design	• South Carolina • Setting not reported	Not Reported	1717 men • 1,211 AAB • 506 White • Mean age 52 71% AAB men	Early Detection	• 1,114 men participated in free prostate screening • 903 men received both a DRE and PSA • Peer-educator and client-navigator interventions significantly associated with obtaining screening for AAB men	• Care coordination • Education/information provision • Empowerment • Direct care provision
Mishel et al., 2002	Test the efficacy of an individualized uncertainty management intervention delivered by telephone to men recently treated for localized PCa directed at managing the uncertainties of their disease and treatment • 3 × 2 randomized block, repeated measures design	• North Carolina • Phone based	Not reported	239 men with PCa • 105 AAB • 134 White • Mean age 64 44% AAB men	Treatment & Survivorship	• Uncertainty management variables indicated significant differences over time • Significant decrease in intensity of treatment side effects symptoms overtime	• Care coordination • Education/information provision • Empowerment • Comfort/emotional support • Direct care provisione
Scura et al., 2004	Evaluate the feasibility of a PCa telephone social support and education intervention to increase the physical, emotional, functional, and interpersonal adaptation of men to PCa • Prospective, RCT	• New Jersey • Phone based	Not reported	17 men with PCa – 10 intervention grp – 7 control grp) • 6 AAB • 10 White • 1 American Indian • Mean age 66 35% AAB men	Treatment & Survivorship	• Telephone counseling resulted in discussions that helped men with the social impact and management of PCa and improved access to supportive services for underrepresented men	• Education/information provision • Comfort/emotional support
Nonzee et al., 2012	Implement and evaluate the efficacy of the Chicago Patient Navigation Research Program (C-PNRP) at a VA hospital, for predominantly low-income minority men with an abnormal PSA • Quasi-experimental intervention design • Study protocol ^a^	• Chicago, IL • Urban Veterans Affairs Hospital	2006—2010	546 men with an abnormal PCa test (245 intervention grp, 245 control grp, 56 in a subsample of surveyed controls) 68% AAB men	Diagnosis, Treatment	^a^ Study protocol – See Simon et al. 2013 for study outcomes Primary outcome measures: - Time to diagnostic resolution - Time to treatment initiation Exploratory/secondary outcome measures - Patient characteristics associated with delay of or non-compliance with diagnostic resolution in intervention arm	• Care coordination • Education/information provision • Empowerment • Comfort/emotional support • Direct care provisions • Advocacy • Logistics assistance
Simon et al., 2013	Evaluate the impact of the Chicago Patient Navigation Research Program (C-PNRP) at a VA hospital on timeliness of diagnostic resolution and treatment initiation among veteran men with an abnormal PCa screening • Quasi-experimental design	• Chicago, IL • Urban Veterans Affairs Hospital	2006—2010	490 men (245 intervention grp, 245 control grp) • 332 AAB • 105 White • 27 Other • 16 race/ethnicity not reported 68% AAB men	Diagnosis, Treatment	• Time to diagnostic resolution • Navigation did not significantly affect time to diagnostic resolution compared to controls (median days of 97 vs. 111) • Time treatment initiation No significant difference in navigated versus control group (median of 93 days vs. 87 days)	• Care Coordination • Education/information provision • Comfort/emotional support • Direct care provision • Logistics assistance ^a^ See ***Nonzee *et al. *2012*** for study protocol
Fleisher et al., 2016	Assess feasibility to address barriers of location and accessibility to PCa risk assessment services in the Prostate Risk, Education & Assessment in the Community with Help (REACH) project among high-risk Black men • Descriptive study	• Northwest Philadelphia, PA (primarily Black neighborhood) • Mobile van at churches or community centers	2009—2011	26 menn • Mean age 52 84.6% AAB men	Early Detection, Diagnosis	• 36 men scheduled for PCa screening REACH; 26 (72%) men showed • 89% (n = 23) used navigation services • 12 of 26 men (46%) had abnormal results • 4 men were unresponsive to follow up (f/u) • 3 of 8 men who received f/u required a biopsy • 2 men were diagnosed with PCa	• Care coordination • Logistics assistance Financial assistance
Dobbs et al., 2020	Investigate whether a patient navigation intervention could improve PCa clinic adherence in a high-risk population • Retrospective case control study	• Chicago, IL • University of Illinois Chicago • Urban, academic urologic oncology clinic	2016—2017	986 men with known or suspected PCa (2,854 scheduled clinic encounters; 1513 Intervention grp, 1341 control grp) Intervention grp: • 59.9% AAB men • 14.6% White • 4.4% Hispanic • 1.8% Asian • 19.4% Unknown/Other/Native American	Diagnosis, Treatment	• 186 (13.9%) missed appointments during control period • 133 (8.8%) missed appointments during intervention period • 36.7% reduction in missed clinical appointments	• Care coordination • Education/information provision • Empowerment • Advocacy

*Cancer Control Continuum, as outlined by the National Cancer Institute – Cancer Control Continuum

**Navigation domains as described in Chan et al. 2023 [11]

*AAB* African American/Black, *PCa* Prostate Cancer, *DRE* Digital Rectal Exam, *PSA* Prostate Specific Antigen, *Grp* Group

^a^ Nonzee et al 2012 [26] and Simon et al 2013 [30] report on the same navigation program but one presents the study protocol and the other presents study outcomes

Study designs used were descriptive [33, 34], quasi-experimental [27, 31, 32] randomized control trial [29], experimental, repeated measures design [29], and a retrospective case control [35]*.* Studies were conducted in the Southern, Midwestern, and Eastern parts of the US with recruitment in the following states: Illinois, New Jersey, Pennsylvania, and North and South Carolina. Navigation services were delivered in person at a veteran’s hospital [27, 31], AAB churches [34], mobile van [33], or over the phone [29, 30, 35]. All but one study was conducted prior to 2012 [34]. Sample sizes ranged from 17 to 1717 participants. AAB men comprised a majority of study participants with only two studies containing less than 50% AAB men [29, 30].

**Fig. 2:**
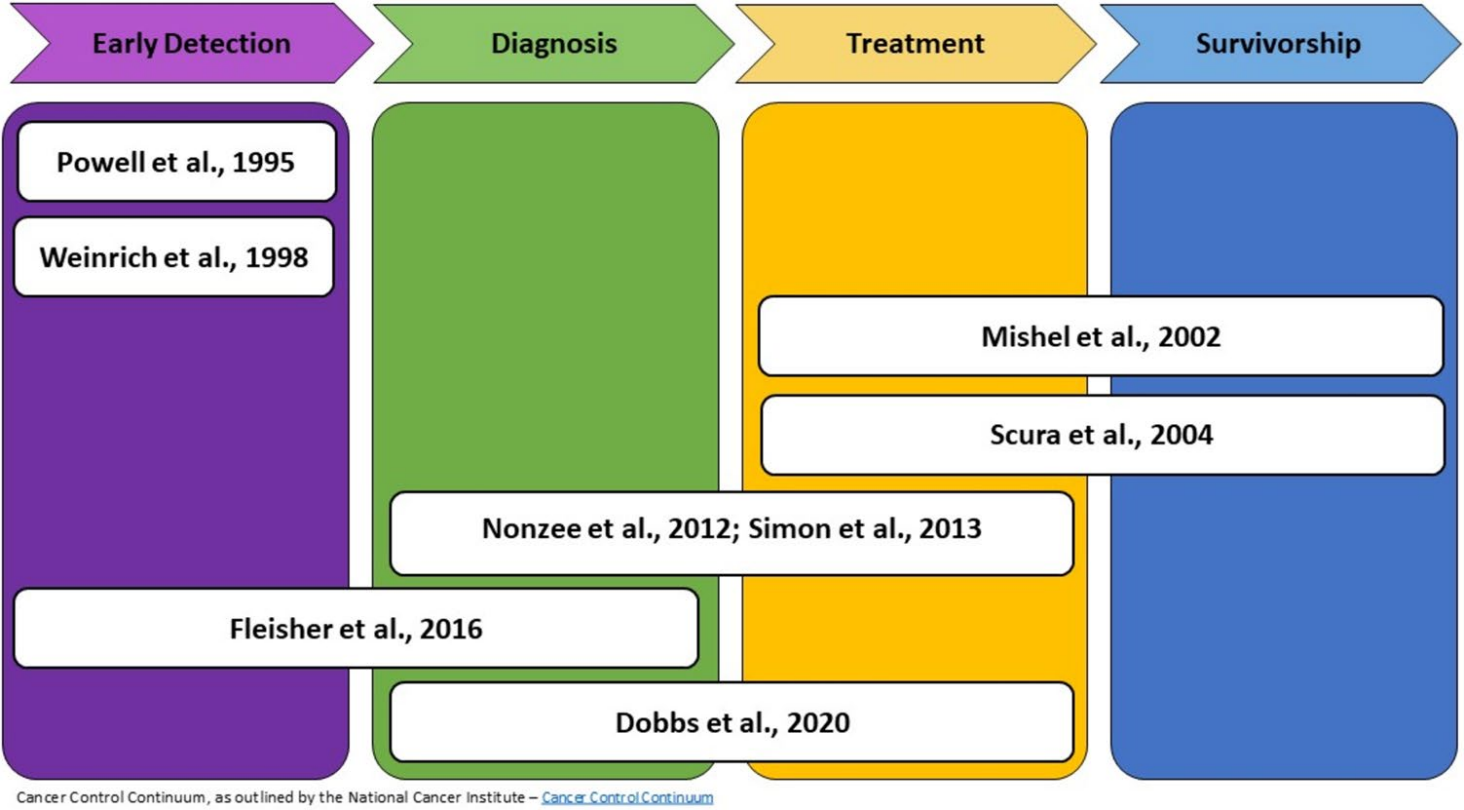
Coverage across the cancer control continuum by study

Figure 2 presents an overview of studies across the cancer control continuum. Three studies provided navigation services during the early detection phase of the cancer continuum [32–34], two studies covering the same navigation program addressed the diagnosis and treatment phase [27, 31] along with a third study [35], and two studies focused on the treatment and survivorship phase [29, 30].

Type of navigation activities provided was organized according to navigation domains outlined by Chan and colleagues [11]. Navigation domains include care coordination, education/informational provision, empowerment, comfort/emotional support, direct care provision, advocacy, logistics assistance, and financial assistance. We defined advocacy as assistance toward resolution or improvement of patient issues within the healthcare system [8]. The most common navigation domains reported were care coordination, education/information provision, and comfort/emotional support.

### Intervention outcomes

Generally, most studies reported beneficial findings between patient navigation and health care outcomes. Three studies reported positive associations between navigation and screening uptake [32–34]. Two studies reported improvements in PCa management, including access to supportive services [29, 30]. One study reported a reduction in missed appointments following navigation [35]. Only one study reported no changes following navigation, with no significant differences in time to resolution in the navigated group versus the control group [31].

### Navigation administration, training, and management

Navigation characteristics are detailed in Table 2. Navigation was provided by social workers [27, 31, 32, 34, 35], nursing staff [30, 32, 34], lay health workers [27, 31], and an oncology research assistant [29]. One study engaged PCa survivors/peers [34]. Two studies included navigator gender and reported male navigators [27, 31]. No study reported the navigator-patient ratio or caseload.

**Table 2:** Navigation characteristics of included studies

Author(s), Publication year, Program name	Navigation objective	Navigator type	Navigator demographics	Navigation site/delivery method/format	Navigation activities	Navigator training
Powell et al., 1995 The Detroit Education and Early Detection Program (DEED)	Culturally specific education to remove attitudinal and behavioral barriers to the health care system, explain the anatomy and function of the prostate, discuss issues and early detections diagnostic methods for PCa	• 6 PCa survivors (ages 49–72) • Nurse coordinator • Social worker	More than 90% of program staff participants were AAB	• In-person after Church service • Telephone	• Education (before PSA test) [delivered by Black male physician & PCa survivor], on PCa, screening, regular physical exams, diet related to PCa progression, prognosis if detected early, digital rectal exam, and trust of the health care system, and sexual function post TX • PCa survivors (peers) answered questions about the emotional toll of cancer, the process and TX effects, & experience with digital rectal exam • All men screened receive a letter with PSA test results • Follow-up phone calls by nurse coordinator for men with elevated PSA test, to make an appointment/follow-up with personal physician, followed prospectively to see if TX sought or reason for no further consultation • Social worker assisted uninsured men with elevated PSA & diagnosed with PCa obtain health insurance	PCa survivors trained on major aspects of PCa
Weinrich et al., 1998 No program name reported	Identify barriers to PCa screening, assist men with navigating health care system	• Social worker • Nurse • Nurse field manager	Not Reported	• Telephone	• Traditional: education program on PCa presented by nurse, PCa handout, Q/A period. Education covered prostate anatomy, early detection, symptoms, and TX options • Peer-educator only: Man of same race shared testimony of PCa screening • Client-navigator only: 1-week after education program, social worker/nurse called men who identified barriers to screening to help them navigate the health care system, included 3 reminders, a calendar, a key ring, and a refrigerator magnet that encouraged prostate checkup • Combination included peer-educator & Client-navigator described above • Nurse field manager followed men with abnormal findings to ensure TX	Not Reported
Mishel et al., 2002 No program name reported	Management of PCa uncertainty and improvement of symptom control directly to patient or patient and family member	Intervention Nurses (matched by ethnicity & gender of patient)	AAB White/Caucasian	• Telephone	Psychoeducational Intervention: • Weekly calls from nurse (for 8 weeks) to assess concerns & uncertainty, followed by cognitive reframing (supporting, validating, and reinforcing), and resources & strategies to help with concerns & encourage patient to take action • Taught patients how to monitor problems, try out solutions & report results during calls • Coached patients to strengthen patient-provider communication (e.g., generating list of questions, how to ask questions, assertive communication skills, etc.) • Mailed printed materials, audiotapes, and videotapes for managing specific problems after each call • Supplemented version (with family member) also received weekly call for 8 weeks with nurse matched by ethnicity and gender, who conducted similar assessments and discussions as patients	Not Reported
Scura et al., 2004 No program name reported	Social and informational support to increase physical, emotional, functional, and interpersonal adaptation to PCa	Oncology research assistant	AAB White/Caucasian	• Telephone	Intervention: • 12 months of telephone social support and education via mailed resource kit • Themes frequently discussed included PCa information seeking, TX choices, leaving a legacy of PCa awareness, coping with disease and TX side effects • Resource kit included a PCa information manual, videotapes, and an audiotape	Not Reported
Nonzee et al., 2012 Chicago Patient Navigation Research Program	Address barriers present at patient-, provider-, practice-, and policy-target levels to patient obtaining follow-up care, and tailor navigation to the identified needs of each patient	• Social worker with an advanced degree • Lay health worker, college-educated	2 Navigators: • AAB • White All Male	• In-person • Telephone	Intervention: • Assess multi-level barriers, and support patient actions • Interact with providers and the health care system on patients’ behalf • Address psychosocial issues • Appointment reminder calls • Link patients with resources • Coordinate transportation • Facilitate patient education & respond to clinical questions • Meet patient face-to-face at clinic visits for social support, and monitor patient progress • Resolve emerging issues or connect patient with VA and community organization resources • Assisted in canceling or rescheduling appointments to avoid delays in diagnostic resolution • Calls for appointment reminders and review procedure-related instructions (e.g., biopsy) • Check-in calls as needed • Navigation intensity varied case by case	• Local, state, and national levels (e.g., annual training conferences sponsored by the National Cancer Institute and the American Cancer Society) • Training topics included exposure to types of patient navigators, development of resource tools for patients, enhancement of communication and collaboration skills • Lead social worker participated in monthly navigation training sessions via the ACS • Research team trained navigators in cultural and communication barriers • Human subjects training, patient education, and electronic medical record training as needed
Simon et al., 2013 Chicago Patient Navigation Research Program (PNRP)	Assist veterans with an abnormal prostate screen proactively identify and resolve personal and systems barriers to care	1 Social worker 1 Lay health worker	2 Navigators: • AAB • White All Male	All Male • Telephone	Intervention: • Navigators conducted face-to-face interviews with patients to identify barriers to care • Follow-up assistance occurred in person or via phone until diagnostic resolution for those without PCa, primary TX completion for those with PCa, or until end of study • Number of contacts varied by need • Men received appointment reminder calls 1-month and 10-days prior to scheduled biopsy to ensure compliance with preparatory instructions, social support, facilitation of patient-provider communication, transportation coordination, and patient education, appointment accompaniment, and connection with a cancer survivor via ACS program	Local training, monthly state-level ACS trainings, national annual PNRP training conferences relative to navigator roles, health disparities, cancer knowledge, cultural diversity, and communication Additional training details reported in protocol (*see Nonzee *et al. *2012, above*)
Fleisher et al., 2016 Prostate Risk, Education and Assessment in the Community with Help (REACH) Program	Address barriers to Black men’s participation in the Prostate Cancer Risk Assessment Program (PRAP) to increase access to education and screening services for men at high risk of PCa	Not Reported	Not Reported	• In-person • Telephone	• Help men overcome barriers (perceived or actual) pertaining to transportation, finances, insurance enrollment paperwork, decision-making issues, or family support • Help uninsured men identify and obtain eligible insurance • Attended every screening event and help participants complete PRAP and navigation informed consent, and explain PRAP screening procedures • Contacted men with abnormal findings to assess need with scheduling, transportation, paying for more test, or other issues	Not Reported
Dobbs et al., 2020 No program name reported	Provide support and advocacy for clinic patients and assistance with patient education and health literacy regarding PCa screening, risks and treatment options	• Clinical social worker with an advanced degree	Not Reported	• Telephone	Intervention: • Navigator attempted phone contact to new patient with known or suspected PCa within 1 week of first appointment - Navigators asked patients about perceived barriers to making their clinical appointment - Provided information, encouragement, and resources to improve clinical adherence • Patients in both cohorts received an automated telephone reminder, 2 days prior to their scheduled visit	• PCa training by clinical staff • Navigator shadowed clinicians to observe prostate biopsy procedures, and discussions regarding the options for elevated PSA and PCa TX options

*AAB* African American/Black, *ACS* American Cancer Society, *NCI* National Cancer Institute, *PCa* Prostate Cancer, *PSA* prostate specific antigen, *TX* Treatment

Nonzee et al., 2012 presents the protocol and Simon et al., 2013 presents outcome data for the same navigation study

Telephone and in-person formats served as the primary delivery methods for navigation. Four studies relied solely on phone-based support [29, 30, 32, 35] and four studies combined modalities, using both phone and in-person strategies to support patients [27, 31, 33, 34].

All of the included studies describe navigator activities, which included follow-up calls by nurse coordinators for elevated PSA tests, appointment reminder calls, assistance with appointment scheduling, and monitoring patient progress. Education, a hallmark of navigation activities, ranged from educational programming on topics such as prostate anatomy and treatment options, to providing patients with an informational resource kit on PCa. Only one study reported an activity categorized as advocacy, described as interacting with providers on patients’ behalf [27]. Examples of navigation activities by domain are detailed in Table 3.

**Table 3:** Example navigation activities in included studies by navigation domain*

Patient Navigation Domains	Example Navigation Activities of Included Studies
Care coordination Refers to instances of patient care coordination between institutions and providers, in addition to direct linkages or referrals to care. Also encompasses administrative actions to coordinate patient care	• Follow-up phone calls by nurse coordinator for men with elevated PSA test, to make an appointment/follow-up with personal physician (Powell et al., 1995) • 1-week after education program, social worker/nurse called men who identified barriers to screening to help them navigate the health care system (Weinrich et al., 1998) • Weekly calls from nurse (for 8 weeks) to assess concerns & uncertainty, and resources & strategies to help with concerns & encourage patient to take action (Mishel et al., 2022) • Check-in calls and face-to-face interviews as needed to assess personal and system barriers, progress, and support patient actions (Nonzee et al. 2012; Simon et al., 2013) • Asked about patients’ perceived barriers to making clinical appointment and provided information, encouragement and resources to improve clinical adherence (Dobbs et al., 2020) • Asked about patients’ perceived barriers to making clinical appointment and provided information, encouragement and resources to improve clinical adherence (Dobbs et al., 2020)
Education/information provision Refers to education or information provided through one-on-one sessions, face to face or by telephone, in group education sessions, or written information and mass media	• Education (before PSA test) on: PCa, screening, regular physical exams, diet related to PCa progression, prognosis if detected early, digital rectal exam, and trust of the health care system, and sexual function post TX (Powell et al., 1995) • Education program on PCa presented by nurse, PCa handout, Q/A period. Education covered prostate anatomy, early detection, symptoms, and TX options. (Weinrich et al., 1998) • Mailed printed materials, audiotapes, and videotapes for managing specific problems after each call (Mishel et al. 2002) • Education via mailed resource kit that included PCa information manual, videotapes, and an audiotape (Scura et al., 2004) • Patient education (Nonzee et al., 2012; Simon et al., 2013) • Provide information and patient education (Dobbs et al., 2020)
Empowerment Refers to personalization of care that aims to empower cancer survivors and support self‐management	• Man of same race shared testimony of PCa screening (Weinrich et al., 1998) • Resources & strategies to help with concerns & encourage patient to take action (Mishel et al., 2002) • Coached patients to strengthen patient-provider communication (e.g., generating list of questions, how to ask questions, assertive communication skills, etc.) (Mishel et al., 2002) • Support patient actions (Nonzee et al., 2012) • Provide encouragement (Dobbs et al., 2020)
Comfort/emotional support Refers to assistance that provides support with emotional well-being	• PCa survivors (peers) answered questions about the emotional toll of cancer, the process and TX effects, & experience with digital rectal exam (Powell et al., 1995) • Weekly calls from nurse (for 8 weeks) to assess concerns & uncertainty, followed by cognitive reframing (supporting, validating, and reinforcing) (Mishel et al., 2002) • 12 months of telephone social support (Scura et al., 2004) • Meet patient face-to-face at clinic visits/appointment accompaniment for social support and address psychosocial issues (Nonzee et al., 2012; Simon et al., 2013)
Direct care provision Refers to support that requires direct clinical expertise	• All men screened receive a letter with PSA test results (Powell et al., 1995) • Nurse field manager followed men with abnormal finds to ensure TX (Weinrich et al., 1998) • Taught patients how to monitor problems (symptoms), try out solutions & report results during calls (Mishel et al., 2002)e • Respond to clinical questions and review procedure-related instructions (e.g., biopsy) (Nonzee et al., 2012; Simon et al., 2013)
Advocacy Refers to improving the healthcare system for patients rather than delivering care to individuals	• Interaction with providers and the health care system on patients’ behalf (Nonzee et al., 2012) *Note: Dobbs *et al*., 2020 noted advocacy but no details were provided*
Language assistance Providing services that address language barriers for patients from non-English backgrounds	None reported
Logistics assistance Support addressing logistical barriers	• Transportation coordination (Nonzee et al., 2012; Simon et al., 2013) • Assess needs and help men overcome barriers to scheduling, transportation, or other issues (Fleisher et al., 2016)
Financial assistance Support addressing financial barriers through available financial aid for patients experiencing financial difficulties or those with unmet financial needs	• Social worker assisted uninsured men with elevated PSA & diagnosed with PCa obtain health insurance (Powell et al., 1995) • Help uninsured men identify and obtain eligible insurance, including barriers to insurance enrollment paperwork (Fleisher et al., 2016) • Assess needs with paying for more tests (Fleisher et al., 2016)

*Navigation domains as described in Chan et al. 2023 [11]; PCa: prostate cancer

Only 3 articles reported navigation training details [27, 31, 35]. Training topics included human subjects training, patient education, health disparities, cancer knowledge, cultural diversity, and communication. Some studies reported using local trainings led by study investigators or clinical staff [27, 31, 35], state level trainings led by the American Cancer Society [27, 35], and national trainings led by the National Cancer Institute [27]. Frequency of training sessions ranged from monthly [27] to annually. Training sessions were delivered in-person, with one article including clinician shadowing to observe PCa-related encounters and discussions [35]. Development of training curriculum and duration of training was not described.

Five out of seven articles reported the racial breakdown of navigators, with AAB and White being the sole categories reported [27, 29–31, 34]. Powell and colleagues were the only study to explicitly describe cultural tailoring [34]. They embedded culturally specific principles by engaging individuals from the community (AAB men) as navigators and implementing the program in a Black church in an AAB community. Cultural context was implicit in other studies—e.g., race-matched community member as navigator or some other role [30, 31, 35].

## Discussion

We examined key characteristics of patient navigation programs for PCa care across the cancer continuum, with a focus on cultural context for AAB men who bear the greatest disease burden in the US. While several systematic reviews have reported on patient navigation among PCa patients [36–38], few navigation programs focused on addressing disparities in PCa faced by AAB men. Although we did not include date limitations, eight published studies are scant given the well-known benefits of patient navigation to address cancer disparities [10, 11, 13, 14], along with the burden experienced by AAB men.

Despite the long history of patient navigation and persistent PCa disparities, all but one study was conducted prior to 2012. Notably, the US Preventive Services Task Force downgraded PCa screening recommendations to a grade D (screening not recommended) in 2012 [39], which was later upgraded to a C in 2018 (offer screening based on considerations) [40]. These changes in screening guidelines may have contributed to implementation of fewer navigation studies, and also contributed to a decline in PCa incidence and an increase in metastatic disease [41]. Given this evolution in screening practices and the fact that AAB men are more likely to be diagnosed at later stages of disease, contemporary efforts are needed to re-engage patient navigation in the early detection phase of PCa and diagnostic resolution to achieve health equity for AAB men.

Navigation programs spanned the cancer control continuum, with more covering the early detection, diagnosis, and treatment phase of care, with fewer in survivorship. Navigation has utility across all phases of the continuum, and recent systematic reviews have noted similar gaps in the literature for navigation during survivorship phase [36, 42]. Navigators can serve as intermediaries with providers and the healthcare system, facilitate patient-provider discussions, ensure patients do not fall through cracks in our healthcare system, and increase enrollment and adherence to clinical trials. There is also a lack of evidence engaging navigation during the palliative care and end-of-life phases, which can be very important for metastatic disease [11].

We wanted to explore the extent to which navigation programs tried to culturally center activities to meet the needs of the most marginalized group in PCa care—AAB men. While only one study explicitly described cultural context or tailoring [34], other studies did consider race-and gender-matched navigators [27, 30, 31]. Dimensions of cultural tailoring or appropriateness of interventions to improve engagement include surface structure and deep structure, which may overlap [43]. Surface structure includes tailoring external components (e.g., settings, staff, and recruitment strategies) to reflect the characteristics of a specific population. Deep structure reflects a deep understanding of cultural norms, practices and beliefs, acknowledges the cultural, social, historical, environmental, and psychological forces that may influence specific racial/ethnic groups different [43]. For example, implementing a support group for AAB men facing PCa that is developed by AAB PCa survivors and led by an AAB facilitator offers a culturally tailored space that supports psychological safety for a group consistently marginalized in the US healthcare system. Previous studies have noted representation matters, particularly in healthcare, as a diverse workforce positively impacts health disparities and can be considered trusted members of the community, particularly cancer survivors who went through the same experience [44–46]. And although not discussed in included studies, navigation should also address linguistic barriers to care (e.g., medical jargon, limited health literacy, and limited language proficiency) to provide language assistance [11, 47]. Furthermore, having solutions to healthcare challenges (e.g., peer navigation) that are co-created with community members and key stakeholders (e.g., community advisory boards) can also enhance cultural tailoring [48–50].

Few of the included studies shared information on navigation training and no studies discussed caseload. This information would be helpful for groups interested in launching similar programs, and effective training practices are critical to the development of sustainable navigation programs, although not well described in the literature [51]. Efforts to standardize training set by the National Navigation Roundtable have identified 7 core competencies of navigation training which include: (i) ethical, cultural, legal, and professional issues, (ii) client and care team interaction, (iii) health knowledge, (iv) patient care coordination, (v) practice-based learning, (vi) systems-based learning, and (vii) communication/interpersonal skills [52]. Improving reporting of navigation training would be helpful to inform what minimum qualifications might be ideal to carryout navigation activities. Additionally, details on ideal or manageable caseloads can inform resources needed for sustainability (e.g., time, number of navigators, and costs) [53].

### Strengths and limitations

We present an extensive overview of patient navigation programs for PCa care within the context of eliminating disparities among AAB men. This review is strengthened by the multidisciplinary review team with expertise in PCa care, cancer navigation, and health equity research, all focused on underserved populations. We also employed a detailed search strategy to ensure a comprehensive review of the literature, transparent reporting to ensure sound methodology, and we focused on an underexplored area within patient navigation—cultural context and appropriateness for AAB men to achieve health equity. Additionally, while we assessed study quality using MMAT that was generally high, we noted experimental trials had lower scores based on ambiguity in the methods, which highlights a need for more detailed reporting. Notable limitations also include publication and citation bias, as it is possible relevant articles with negative results were not published and excluded from this review [54]. Additionally, our exclusion criteria (e.g., outside the US and sample size of AAB men less than 30%) may contribute to a gap in what is known about navigation in PCa care in general or in other underserved populations (e.g., Latino/Hispanic patients), and may not reflect all navigation elements that could support AAB men facing PCa.

## Conclusion

Navigation programs in PCa care across the cancer care continuum remain beneficial; however, gaps remain to meet the needs of AAB men facing PCa to achieve health equity. Contemporary navigation programs are needed to address disparities among AAB men in PCa care, given it represents one the largest of all cancer disparities. Additionally, more research and details are needed on navigation activities, including necessary training, and management for more consistency in the widespread implementation of best practices.

## Supplementary Information

Below is the link to the electronic supplementary material.

ESM 1 (DOCX 107 KB)

ESM 2 (PDF 93.5 KB)

## Data Availability

Data is provided within the manuscript or supplementary information. Excel spreadsheets generated for data synthesis during the current study are available from the corresponding author upon request.
