# Supplementary material for: Addressing barriers to care for African American men facing prostate cancer: a scoping review of navigation programs
Source: Support Care Cancer. 2025 Oct 14;33(11):947. doi: 10.1007/s00520-025-10009-7 (PMC12521313; doi:10.1007/s00520-025-10009-7)
Supplement: Supplementary file 2 — (PDF 93.5 KB) [file 520_2025_10009_MOESM2_ESM.pdf]

APPENDIX: Search Strategy for Patient Navigation Scoping Review

DATABASE	SEARCH STRATEGY
PubMed	(“Prostatic Neoplasms”[mesh] OR “prostate cancer” OR “prostate gland cancer” OR “prostatic cancer”) AND (“Patient Navigation”[mesh] OR “patient navigation” OR “patient navigator” OR “navigation program” OR “navigation programs” OR “navigation programme” OR “peer navigation” OR “peer navigator” OR “cancer navigation” OR “navigation system” OR “navigation intervention” OR “Health Education”[mesh] OR “health education” OR “cancer education” OR “cancer communication” OR “health advisor” OR “health advisors” OR “Community Health Workers”[mesh] OR “community health workers” OR “Health Promotion”[mesh] OR “health promotion” OR “outreach program” OR “outreach programs” OR “outreach programme” OR “outreach programmes” OR “Health Status Disparities”[mesh] OR “health status disparities” OR “healthcare disparities” OR “health care disparities” OR “health disparities” OR “Community-Based Participatory Research”[mesh] OR “research participation” OR “Health Knowledge, Attitudes, Practice”[mesh] OR “health attitudes” OR “health knowledge” OR “cancer knowledge” OR “medically underserved” OR “Early Detection of Cancer”[mesh] OR “early detection” OR “cancer detection” OR “cancer screening” OR “decision-making” OR “informed decision”) AND ("Black or African American"[Mesh] OR African American* OR black men OR black males)
Web of Science	(“Prostatic Neoplasms” OR “prostate cancer” OR “prostate gland cancer” OR “prostatic cancer”) AND (“patient navigation” OR “patient navigator” OR “navigation program” OR “navigation programs” OR “navigation programme” OR “navigation programmes” OR “peer navigation” OR “peer navigator” OR “cancer navigation” OR “navigation system” OR “navigation intervention” OR “health education” OR “cancer education” OR “cancer communication” OR “health advisor” OR “health advisors” OR “community health workers” OR “health promotion” OR “outreach program” OR “outreach programs” OR “outreach programme” OR “outreach programmes” OR “health status disparities” OR “healthcare disparities” OR “health care disparities” OR “health disparities” OR “research participation” OR “health attitudes” OR “health knowledge” OR “cancer knowledge” OR “medically underserved” OR “early detection” OR “cancer detection” OR “cancer screening” OR “decision-making” OR “informed decision”) AND (African American* OR black men OR black males)
Embase	('prostate cancer'/exp OR 'prostate cancer' OR 'prostate tumor'/exp OR 'prostate tumor' OR 'prostate gland cancer'/exp OR 'prostate gland cancer' OR 'prostatic neoplasms'/exp OR 'prostatic neoplasms') AND ('patient navigation'/exp OR 'patient navigation' OR 'patient navigator'/exp OR 'patient navigator' OR 'navigation program' OR 'navigation programs' OR 'navigation programme' OR 'navigation programmes' OR 'peer navigation' OR 'peer navigator' OR 'cancer navigation' OR 'navigation system'/exp OR 'navigation system' OR 'navigation intervention' OR 'health education'/exp OR 'health education' OR 'cancer education'/exp OR 'cancer education' OR 'cancer communication' OR 'health advisor' OR 'health advisors' OR 'community health

	workers'/exp OR 'community health workers' OR 'health auxiliary'/exp OR 'health auxiliary' OR 'health promotion'/exp OR 'health promotion' OR 'outreach program' OR 'outreach programs' OR 'outreach programme' OR 'outreach programmes' OR 'health disparity'/exp OR 'health disparity' OR 'research participation'/exp OR 'research participation' OR 'attitude to health'/exp OR 'attitude to health' OR 'health knowledge'/exp OR 'health knowledge' OR 'cancer knowledge' OR 'medically underserved'/exp OR 'medically underserved' OR 'early cancer diagnosis'/exp OR 'early cancer diagnosis' OR 'early detection' OR 'cancer detection'/exp OR 'cancer detection' OR 'cancer screening'/exp OR 'cancer screening' OR 'decision making'/exp OR 'decision making' OR 'informed decision making'/exp OR 'informed decision making' OR 'informed decision') AND ('african american'/exp OR 'african american' OR 'black men' OR 'black males' OR 'black'/exp OR black OR 'blacks'/exp OR blacks OR 'black person'/exp OR ‘black person’)
CINAHL Complete	(“Prostatic Neoplasms” OR “prostate cancer” OR “prostate gland cancer” OR “prostatic cancer”) AND (“patient navigation” OR “patient navigator” OR “navigation program” OR “navigation programs” OR “navigation programme” OR “navigation programmes” OR “peer navigation” OR “peer navigator” OR “cancer navigation” OR “navigation system” OR “navigation intervention” OR “health education” OR “cancer education” OR “cancer communication” OR “health advisor” OR “health advisors” OR “community health workers” OR “health promotion” OR “outreach program” OR “outreach programs” OR “outreach programme” OR “outreach programmes” OR “health status disparities” OR “healthcare disparities” OR “health care disparities” OR “health disparities” OR “research participation” OR “health attitudes” OR “health knowledge” OR “cancer knowledge” OR “medically underserved” OR “early detection” OR “cancer detection” OR “cancer screening” OR “decision-making” OR “informed decision”) AND (African American* OR black men OR black males)
